# Supporting youths in global crises: an analysis of risk and resources factors for multiple health complaints in children and adolescents during the COVID-19 pandemic

**DOI:** 10.3389/fpubh.2025.1510355

**Published:** 2025-02-13

**Authors:** Karoline Habermann, Ann-Kathrin Napp, Franziska Reiß, Anne Kaman, Michael Erhart, Ulrike Ravens-Sieberer

**Affiliations:** ^1^Department of Child and Adolescent Psychiatry, Psychotherapy, and Psychosomatics, Research Section “Child Public Health”, University Medical Center Hamburg-Eppendorf, Hamburg, Germany; ^2^Department of Health and Education, Alice Salomon University of Apllied Science Berlin, Berlin, Germany

**Keywords:** youths, health complaints, global crises, COPSY, family

## Abstract

**Introduction:**

The number of global crises increased in recent years, significantly affecting the health and well-being of children and adolescents worldwide. Awareness of these global crises and their impact on health and well-being has risen since the COVID-19 pandemic. Research indicates that some children and adolescents are more vulnerable to the challenges resulting from these crises than others. This study examines the risk and resources factors for multiple health complaints (MHC) in children and adolescents in Germany during the COVID-19 pandemic.

**Methods:**

We analyzed data from the German population-based longitudinal COPSY (COvid-19 and PSYchological Health) study. A total of 1,673 children and adolescents aged 11 years and older and their parents participated in at least one of the five survey waves between 2020 and 2022. We assessed MHC using the international Health Behavior in School-aged Children Symptom Checklist (HBSC-SCL), which includes four somatic complaints (e.g., headache, abdominal pain) and four psychological complaints (e.g., feeling low, sleeping difficulties). We conducted a mixed model panel regression analysis to examine longitudinal changes in MHC and identified sociodemographic, psychosocial, and pandemic-related risk factors and resources.

**Results:**

Our results revealed increased MHC throughout the COVID-19 pandemic. Girls, single-parent children and adolescents, as well as children and adolescents of mentally ill or highly burdened parents, were at particular risk. A higher subjective perceived burden of the pandemic and COVID-19-related worries were significantly associated with a higher level of MHC. Personal resources, a positive family climate, and more perceived social support were significantly associated with a lower level of MHC.

**Discussion:**

This paper highlights the vulnerability of children and adolescents in a global crisis, such as the COVID-19 pandemic. The results provide a scientific foundation for targeted health promotion and intervention strategies to protect and maintain the health and well-being of young people in future crises.

## 1 Introduction

Health complaints, such as headache, abdominal pain, feeling low, or sleeping difficulties are widely common in childhood and adolescence. In fact, it is not rare that two or more health complaints occur at the same time, a phenomenon referred to as “Multiple Health Complaints” (MHC). International studies suggest that one in three children and adolescents experiences MHC (1). Following a holistic approach, MHC include somatic and psychological health complaints and their reciprocal interferences. MHC are self-reported health symptoms that are often medically unexplained and known to be a relevant indicator of mental health and well-being among children and adolescents (1, 2). Severe and multiple complaints can negatively affect the quality of life in many aspects and limit daily functioning (3). Suffering from MHC can have a negative impact not only on children’s and adolescents’ psychosocial development, academic performance, and social participation but also on their mental health status later in adulthood (4–6). Being affected by MHC is significantly associated with negative life events and a higher level of perceived stress in everyday life (7, 8). According to the *perseverative cognition hypothesis*, a preoccupation with stress combined with a perception of uncontrollability increases the likelihood of experiencing somatic symptoms through physical activation (9).

Children and adolescents in the twenty-first century are growing up in times of multiple global crises and are constantly exposed to the challenges resulting from these crises (10–12). Among other things, they deal with the worldwide pandemic with severe social and psychological consequences, an increasing number of natural disasters due to climate change, as well as inflation and current wars.

The Coronavirus disease 2019 (COVID-19) pandemic was one of the major sociohistorical disruptions of everyday life in the twenty-first century and continues to have a profound impact on many aspects of society, including mental and physical health (13). Epidemiological studies showed that the direct effects of acute COVID-19 are less severe in children and adolescents than in adults (14, 15), and children and adolescents are more likely to recover without long-term consequences (16, 17). At the same time, children and adolescents were affected by extensive public health measures to prevent infection, such as social distancing rules, complete closure of daycare centers and schools, home confinement, and massive restrictions on their leisure time activities with closed culture institutions and stores (except for grocery and drugstores). These restrictions in everyday life presented severe challenges for their psychosocial adjustment and had a significant impact on their mental health (18). Research shows higher prevalence rates of low health-related quality of life (HRQoL) and mental health problems, specifically of internalizing symptoms, such as anxiety and depression (19, 20), as well as MHC among children and adolescents during the COVID-19 pandemic (21–23).

The specific impact of different crises such as climate and war-related stress on the health and well-being of children and adolescents is less well studied. However, first evidence indicates a negative effect of global crises on the mental health of children and adolescents. Studies show that children and adolescents are increasingly concerned about these challenges (21, 24–26). As adolescence is a critical period in life for the development of human potential, children and adolescents are confronted with the uncertainties of cumulative global crises at a very vulnerable time in their lives (27).

Further research indicates that some children and adolescents are more vulnerable than others. It is essential to understand the underlying risk factors and resources in order to explain individual differences in adaptation and long-term health outcomes of children and adolescents (26, 28). Meta-analyses, focusing on the COVID-19 pandemic found higher prevalence of mental health problems in girls compared to boys and adolescents compared to younger children (20, 29, 30). Previous work has identified a low socioeconomic status of the family, parental mental health problems, and family functioning as risk factors for poor mental health (31). Further, children and adolescents with pre-existing mental or physical health problems appeared to be more vulnerable to aggravating health problems during the COVID-19 pandemic (32). On the contrary, resources, such as an optimistic attitude, a better parent–child relationship, and more perceived familial and social support were found to facilitate better coping with the pandemic among children and adolescents (21, 33).

Several studies already exist that examined risk and resources factors associated with mental health and well-being among children and adolescents during the COVID-19 pandemic. To our knowledge, there are, however, currently no studies that specifically focus on risk and resources factors associated with MHC. Consequently, this study aims to identify risk factors as well as resources for MHC among children and adolescents during the COVID-19 pandemic as an example of a global crisis in the twenty-first century. We expect that socially disadvantaged children and adolescents are more vulnerable to MHC. Furthermore, we assume that a higher perceived burden of a crisis situation such as the COVID-19 pandemic and COVID-19-related worries are associated with a higher level of MHC. On the other hand, we expect that personal resources, such as an optimistic attitude toward the future, higher self-efficacy, and the perception of sufficient problem-solving skills, as well as familial and social resources, are associated with a lower level of MHC. Familial resources may include a good family climate with a high level of perceived family support and strong familial relationships. Social resources may be characterized by more perceived social support.

## 2 Materials and methods

### 2.1 Study design and sample

The German population-based longitudinal COPSY (COVid-19 and PSYchological Health) study monitors mental health and HRQoL among children and adolescents during the COVID-19 pandemic in Germany in five survey waves (W1–W5). The first wave (W1) took place from May to June 2020, when Germany was under a partial lockdown with preventive distancing measures and limited contact options in place. The second survey wave (W2) was conducted from December 2020 to January 2021 at the beginning of the second nationwide lockdown in Germany with a complete closure of daycare centers and schools as well as all cultural institutions and stores (except for grocery and drugstores) for almost 6 months due to high infection rates and a high percentage of hospitalized cases. The third wave (W3) collected data from September to October 2021 after some months with low infection rates and loosened restrictions during the summer. The fourth wave (W4) was undertaken in February 2022 at the end of the second winter with high infection and hospitalization rates. The last wave (W5) was conducted from September to October 2022, after the third summer of the COVID-19 pandemic, with lower infection and higher vaccination rates and without any preventive distancing measures in place (34, 35).

We invited families to participate in the COPSY study via an online survey using quota sampling. This method ensured that the sample reflects the sociodemographic characteristics of the German population. Families were re-invited at each follow-up. To ensure comparability across all five waves, we compensated for dropouts by recruiting additional families at each survey wave. We collected parent-reported data for children and adolescents aged 7–17 years and self-reported data from children and adolescents aged 11 years and older. *N* = 2,471 families participated in at least one survey wave of the COPSY study, including *n* = 1,673 self-reports of children and adolescents. Previous publications of the COPSY study describe the exact sample sizes and design of the five survey waves (W1–W5) (36–39). The longitudinal response rate over the five survey waves was 86.1%. The COPSY study was approved by the Local Psychological Ethics Committee (LPEK-0151) and the Commissioner for Data Protection of the University of Hamburg.

### 2.2 Measures

#### 2.2.1 Sociodemographics

We assessed the age and gender of children and adolescents and their parents via self-reports. The parents also provided information on their education and occupation, single parenthood, living space of the family, and migration background. To evaluate parental education, we requested information on the highest academic and vocational qualifications of both parents. The parents were categorized into three groups (low, medium, and high education level) according to the international “Comparative Analysis of Social Mobility in Industrial Nations” (CASMIN) (40).

#### 2.2.2 Multiple health complaints

We assessed MHC with the Health Behavior in School-aged Children Symptom Checklist (HBSC-SCL) (41, 42). The HBSC-SCL is a non-clinical, internationally well-established, and validated instrument to measure the frequency of MHC in children and adolescents. It has shown acceptable unidimensionality, internal consistency, test–retest reliability, and international comparability (43, 44). The HBSC-SCL includes eight complaints: four physical (headache, abdominal pain, backache, and dizziness) and four psychological (feeling low, irritability, feeling nervous, and sleeping difficulties). Children and adolescents were presented with five options to describe the frequency of each health complaint (1: never, 2: 1–2 times per week, 3: 3–4 times per week, 4: 5–6 times per week, 5: daily). To reflect the short-term changes in the COVID-19 pandemic, we changed the reference period to “within the last week,” instead of “within the last 6 months.” We calculated the unweighted sum score of the eight items, ranging from 8 to 40, with higher values indicating more complaints. Internal consistency at each wave was good (Cronbach’s alpha (α) > 0.80: W1 = 0.83, W2 = 0.86, W3 = 0.86, W4 = 0.87, W5 = 0.86). To assess the relative frequency of each health complaint at each measurement point, we divided the participants into groups of subjects who experienced each complaint “at least on half of the days per week” (≥3–4 times per week) vs. those who experienced it “less frequently.” Based on the cut-off used in the HBSC study (42), we calculated a cut-off, which reflects the prevalence of MHC among children and adolescents within the last week (cut-off = “two or more health complaints on more than half of the days per week”).

#### 2.2.3 Risk and resources factors

In addition to the sociodemographic factors mentioned above, we assessed parental mental health as an established psychosocial risk factor for children’s and adolescents’ mental health during the COVID-19 pandemic (45). Therefore, a question asked the parents to indicate whether they had a mental disease or mental health problems diagnosed by a physician or psychotherapist. To understand pandemic-related risk factors, parents were asked to provide information on whether there has been a COVID-19 infection in the family and whether the child was infected. Children and adolescents, as well as their parents, further provided information about their subjective perceived burden of the pandemic at each survey wave (1: not stressful at all – 5: highly stressful). Additionally, children and adolescents rated their worries about infection with COVID-19 at each measure point (1: not worried at all – 5: highly worried).

We assessed personal resources with the Personal Resources Scale (PRS), which includes five items asking for a self-evaluation of problem-solving skills, self-efficacy, and optimism for the future (1: disagree – 4: total agree) (46). Family climate, as an indicator of familial resources, was evaluated with four items from the Cohesion subscale of the Family Climate Scale (FCS), asking about perceived positive familial interaction and support (1: disagree – 4: total agree) (47). Social resources characterized by more perceived social support were measured with four items of the Social Support Scale (SSS), which inquired about having enough conversation partners and feeling integrated into a positive social environment (1: never – 5: always) (48). We calculated sum scores for all three scales, with higher values indicating more perceived resources (PRS [5–20], FCS [4–16], SSS [4–20]).

#### 2.2.4 Data analysis

For each survey wave, we calculated the absolute and relative frequencies of the eight health complaints of the HBSC-SCL, the HBSC-SCL sum score, and the described cut-off. Gender differences are expressed as odds ratios with 95%-confidence intervals (OR [CI]).

We conducted repeated measures ANOVAs to examine the variation in the HBSC-SCL sum scores, the cut-off, and the eight health complaints across the five survey waves. We performed a mixed model panel regression analysis with random effects to investigate the longitudinal changes in MHC during the COVID-19 pandemic. This analysis aimed to identify relevant risk factors and resources. As predictors, we estimated coefficients, representing an effect of time during the pandemic, time-constant factors (age, gender, migration background, single parenthood, low parental education, less living space, parental mental problems), time-varying factors (parental subjective perceived burden of the pandemic, children’s subjective perceived burden of the pandemic, children’s COVID-19 related worries, reported previous infection with COVID-19) and resources factors (personal resources, family climate, social support). The random effects model incorporates a random intercept for each participant, allowing for the simultaneous inclusion of time-variant and time-invariant predictors.

The data of each survey wave was adjusted to match the sociodemographic characteristics of the German population. This adjustment was based on the Microcensus 2018 for waves W1 to W3 and Microcensus 2020 for waves W4 and W5. We conducted a power analysis using G-Power 3.1 software (49). Based on the parameters for statistical significance of *p* (α) *<* 0.05 and a power of 95% for a small effect (*f* = 0.1) between the survey waves W1 to W5 (within factor) and between two groups (between factor), and interaction between survey waves W1–W5 and two groups (within between interaction), this analysis determined a minimum sample size of *n* = 782 participants. For the panel analyses, the PLM package by Croissant and Millo (50) in R was used (Version R 4.3.0). All other analyses were carried out using SPSS (Version 29).

## 3 Results

### 3.1 Sociodemographics

In total, *N* = 1,673 children and adolescents aged 11 years or older and their parents participated in at least one survey wave (*M*_age_ = 15.23, *SD* = 2.56; 52.1% female). The majority of the participants (82.9%) had no migration background. Furthermore, 82.5% of the parents reported at least a medium education level, 53.1% were full-time employed, and 20.0% were single parents. Table 1 provides information on the sociodemographic characteristics of the sample.

**Table 1:** Details on the sociodemographic characteristics of the COPSY sample (*N* = 1,673).

	*n* (%)	*M* (SD)
**Age child**				15.23 (2.56)
11–13 years	494 (29.5)	
14–17 years	807 (48.2)	
≥18 years	372 (22.2)	
**Gender child**		
Female	864 (51.6)	
Male	796 (47.6)	
Diverse	13 (0.8)	
**Age parent**				46.34 (7.22)
**Gender parent**		
Female	931 (55.6)	
Male	740 (44.2)	
Diverse	2 (0.1)	
**Migration background**		
No	1,376 (82.9)	
Yes	283 (17.1)	
**Parental education (CASMIN-Score)**		
Low	291 (17.5)	
Medium	959 (57.5)	
High	417 (25.0)	
**Single parent**		
No	1,338 (80.0)	
Yes	335 (20.0)	
**Occupation parent**		
Full-time employed	891 (53.3)	
Part-time employed	485 (29.0)	
Self-employed	71 (4.2)	
Other employment	29 (1.7)	
Stay-at-home parent	97 (5.8)	
Retiree/pensioner	47 (2.8)	
On parental leave	14 (0.8)	
Unemployed	39 (2.3)	

Unweighted data. M = mean; SD = standard deviation. Reported data at the last time of participation.

### 3.2 Multiple health complaints

**Figure 1:**
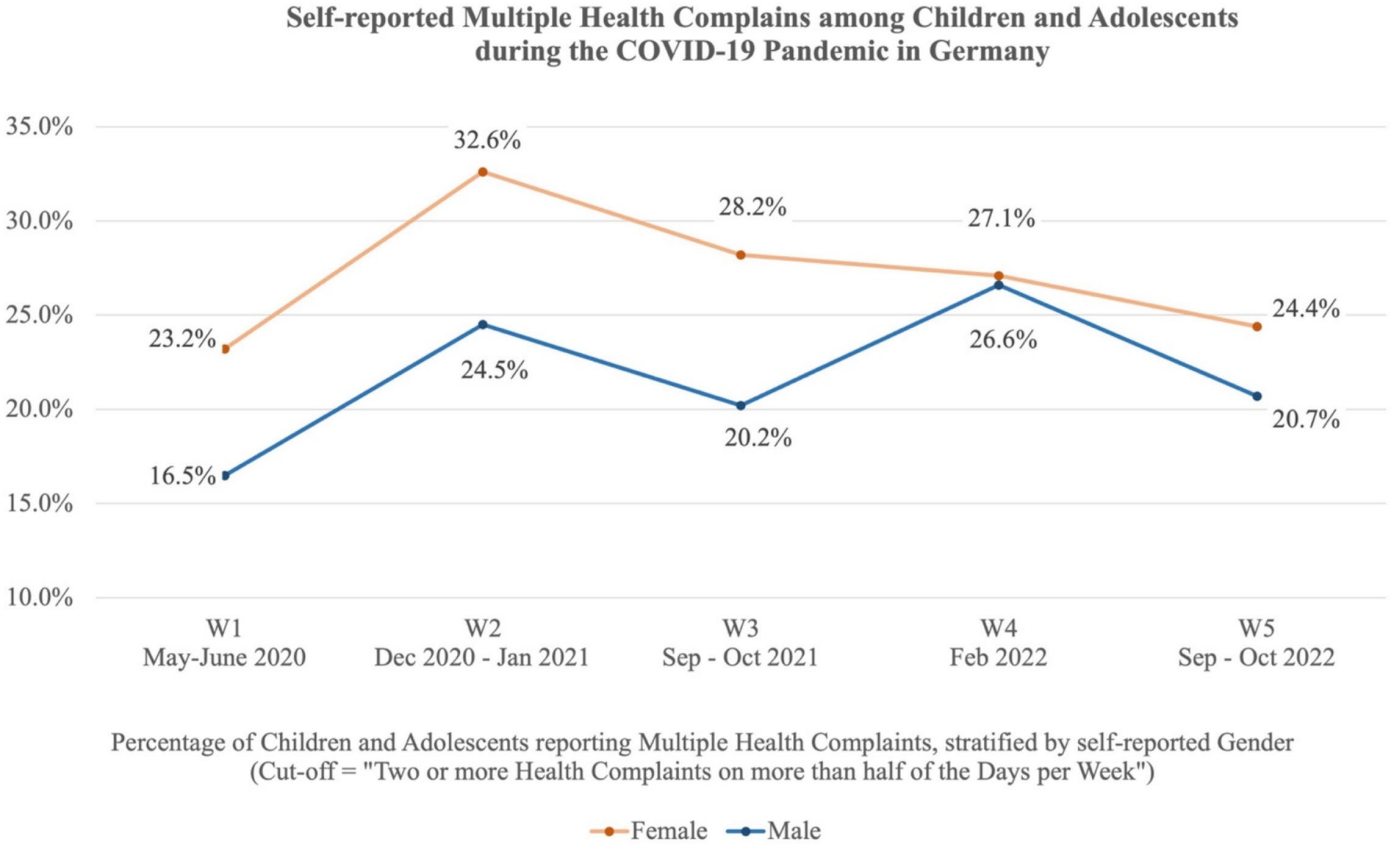
Self-reported MHC among children and adolescents in Germany during the COVID-19 pandemic. The cut-off reflects the percentage of children and adolescents reporting two or more health complaints on more than half of the days per week. The stratification is based on self-reported gender.

Figure 1 shows the self-reported MHC among children and adolescents during the COVID-19 pandemic in Germany, stratified by gender. The cut-off reflects the percentage of children and adolescents experiencing two or more health complaints on more than half of the days within the last week. The highest prevalences were found in the winter of 2020/21 with 28.5% (W2) of the children and adolescents reporting MHC, and in the winter of 2022 with 26.8% (W4). In autumn 2022, the prevalence of MHC had decreased to 22.1% (W5) but was still higher than at the beginning of the pandemic (19.8% in May–June 2020 (W1)). Girls reported a significantly higher level of MHC compared to boys in W1 (OR = 1.53 [1.12–2.09]), W2 (OR = 1.49 [1.14–1.95]), and W3 (OR = 1.55 [1.18–2.04]). No significant gender differences were found for survey waves W4 and W5. The repeated measures ANOVAs with a Greenhouse correction showed significant differences between the five survey waves, *F* (3.61, 1567.93) = 7.47, *p* < 0.001, partial *η*^2^ = 0.017. Supplementary material S1 shows the HBSC-SCL sum scores for each survey wave, stratified by gender.

The most common somatic symptom was headache, with the highest prevalence in the winter of 2020/2021 (16.4%) and in the winter of 2022 (15.5%). The most frequently reported psychological health complaint was sleeping difficulties. Sleeping difficulties were most prevalent at the beginning of the pandemic, with about one out of five children reporting sleeping difficulties on more than half of the days per week. Repeated measures ANOVAs with a Green-house correction showed significant variation between the five measurement points for seven of the eight health complaints (headache: *p* < 0.001, abdominal pain: *p* < 0.001, backache: *p* = 0.004, dizziness: *p* = 0.012, feeling low: *p* < 0.001, irritability: *p* = 0.001, nervousness: *p* = 0.065, sleeping difficulties: *p* < 0.001). Supplementary material S2 shows the relative frequencies of each health complaint reported by children and adolescents on more than half of the days per week across the five survey waves.

### 3.3 Risk and resources factors

Table 2 shows the included predictors in the panel linear mixed model with random effects and the results. The parameter estimates indicate the association between the different predictors with a higher level of MHC, controlling for the other predictors by simultaneous inclusion of all parameters. Time during the pandemic was significantly positively associated with a higher level of MHC (+0.17, *p* < 0.001). In terms of time-constant sociodemographic risk factors, female gender (+0.78, *p* < 0.001) and single parenthood (+0.66, *p* < 0.01) were significantly associated with a higher level of MHC. We identified parental mental problems (+0.74, *p* < 0.01) as a psychosocial risk factor that was significantly positively associated with a higher level of MHC among children and adolescents. In terms of time-varying pandemic-related risk factors, the subjective perceived burden of the pandemic among children and adolescents (+0.52, *p* < 0.001) as well as that among the parents (+0.22, *p* < 0.01) and being more worried about an infection with COVID-19 (+0.33, *p* < 0.001) were significantly associated with a higher level of MHC. An infection with COVID-19 was no significant risk factor in this model. Looking at the resources of children and adolescents, more personal resources (−0.47, *p* < 0.001), a more positively perceived family climate (−0.32, *p* < 0.001), and more social support (−0.21, *p* < 0.001) were significantly associated with less MHC. The overall model fit was *R*^2^ = 0.31.

**Table 2:** Predictors of MHC among children and adolescents.

	**Estimate**	**Std. Error**	***z*-value**	***p***	
(Intercept)	22.19	0.86	25.68	<0.001	***
**Effect of time**						0.17	0.04	4.25	<0.001	***
**Time-constant risk factors**					
Age	0.05	0.03	1.49	0.14	
Female gender	0.78	0.16	4.85	<0.001	***
Migration background	0.30	0.23	1.33	0.18	
Higher parental education (CASMIN)	−0.16	0.12	−1.33	0.18	
Single parenthood	0.66	0.19	3.44	<0.01	**
Living space (m^2^ per person)	−0.01	0.01	−1.08	0.28	
Parental mental problems	0.74	0.23	3.19	<0.01	**
**Time-varying risk factors**					
Parental perceived burden of the pandemic	0.22	0.07	3.12	<0.01	**
Subjective perceived burden of the pandemic	0.52	0.06	8.10	<0.001	***
Worries about an COVID-19 infection	0.33	0.05	7.12	<0.001	***
Infection with COVID-19	−0.14	0.15	−0.92	0.36	
**Resources**					
Personal resources	−0.47	0.02	−19.06	<0.001	***
Family climate	−0.32	0.03	−10.07	<0.001	***
Social support	−0.21	0.02	−8.57	<0.001	***

Higher values indicate a higher level of MHC. *R*^2^ = 0.31; ***p < 0.001; **p < 0.01.

In terms of pandemic-related risk factors, the subjective perceived burden of the pandemic among children and adolescents and COVID-19-related worries were more prominent at the beginning of the pandemic. Girls were more likely to report feeling burdened by the pandemic in W1 (OR = 1.35 [1.03–1.74]) and W2 (OR = 1.57 [1.14–2.16]) compared to boys. In W3, W4, and W5, we found no significant gender differences. Supplementary materials S3 and S4 illustrate gender-specific data on the subjective burden of the pandemic and worries about COVID-19 infection.

## 4 Discussion

The present paper investigates MHC among children and adolescents during the COVID-19 pandemic in Germany using data from the German population-based longitudinal COPSY study. The purpose was to identify significant risk and resources factors for MHC using the COVID-19 pandemic as an example of a global crisis in the 21st century. This knowledge is relevant to support children and adolescents with targeted intervention and prevention programs in times of multiple global crises.

### 4.1 Multiple health complaints

Our study reveals that time during the pandemic is significantly positively associated with a higher level of MHC. We found higher prevalence rates in particular during the winters of 2020/2021 (W2) and 2022 (W4). At these measurement points, more than one out of four children and adolescents reported experiencing two or more health complaints on at least half of the days per week. We found an improvement in MHC in the third year of the COVID-19 pandemic, which can be explained by ongoing adaptation processes leading to increased resilience, the normalization of everyday social and family life due to fewer restrictions, and the decrease in serious cases due to the availability of effective vaccines. Nevertheless, the prevalence of MHC in autumn 2022 is still higher compared to the beginning of the pandemic. Comparable national data from the German HBSC study indicates a rise in the prevalence of MHC among children and adolescents over the last years, from 26.3% in 2018 to 41.7% in 2022, referring to the last 6 months (51, 52). On an international level, a meta-analysis from Potrebny et al. showed an increase in health complaints among children and adolescents over the last four decades (53). This trend seemed to accelerate during the COVID-19 pandemic. The three most recent survey waves of the international HBSC study found an increasing rise in MHC among children and adolescents. In 2014, 33% of children and adolescents aged 11–15 reported experiencing MHC more than once a week during the last 6 months. This percentage was 36% in 2018 (+3 percentage points compared to 2014) and further increased to 44% in 2022 (+8 percentage points compared to 2018 and + 11 percentage points compared to 2014) (54). Consequently, if MHC among children and adolescents increase during a global crisis such as the COVID-19 pandemic, it is necessary to raise awareness for the early detection of MHC among parents and caregivers. Screening for children and adolescents with MHC in pediatric outpatient clinics or other healthcare facilities opens the possibility of identifying affected children and adolescents and enrolling them in prevention and intervention programs.

### 4.2 Risk and resources factors

Looking at sociodemographic risk factors for MHC, our study reveals a significant gender difference in the prevalence of MHC among children and adolescents. Girls reported significantly higher levels of MHC and a substantially higher subjective burden, especially at the beginning of the pandemic. Several studies describe the vulnerability of girls during the COVID-19 pandemic (18, 20, 31). Recent research indicates that the disparity in MHC between genders has grown, especially in the last few years (54). These gender differences may be due to different socialization practices encouraging girls to express their emotional experiences more openly than boys (55) or an increased sensitivity to stress due to hormonal changes during puberty (56). Under the influence of hormonal changes (57), the gender difference in experience and consequences of stressful life events is particularly pronounced in adolescence (58). Additionally, girls and boys use different coping strategies when dealing with stressful situations (59). Gender-sensitive strategies are therefore necessary to support children and adolescents in times of global crises.

Furthermore, our study reveals that children and adolescents from single-parent families reported significantly higher levels of MHC. These families face cumulative socioeconomic, psychosocial, and environmental burdens (60) and have a higher risk for mental health problems (18). In addition, single parenthood is associated with lower household income and socioeconomic status, especially in a global pandemic with severe social and economic impact (61). Other sociodemographic risk factors, which are also associated with a lower socioeconomic status, such as lower parental education, migration background, or living space per person, were not significantly associated with MHC in this study. Nevertheless, further research indicates that the COVID-19 pandemic had unequal effects, particularly affecting people in vulnerable situations (61). To effectively support children and adolescents in a global crisis, it is crucial to identify burdened and particularly vulnerable groups such as single-parent families and provide them with targeted support programs.

Looking at psychosocial risk factors for MHC, we found that parental mental health problems and a higher perceived parental burden of the pandemic had a significant adverse effect on MHC among children and adolescents. Parental mental health problems and high levels of parental stress are closely related to their parenting skills (62). The way parents raise their children, their coping strategies, and the overall family environment significantly affect the well-being of children and adolescents during and for a long time after a stressful life event (63). Consequently, enhancing parental emotional stability and parenting competence can positively influence the health and well-being of children and adolescents. Therefore, parents represent an important target group for prevention and intervention, including parenting programs or family counseling.

In addition to the higher perceived parental burden of the pandemic, we found a higher perceived burden of the pandemic among children and adolescents and COVID-19-related worries as significant pandemic-related risk factors for MHC. Dealing with distancing rules, school closures, homeschooling, and restrictions on leisure time activities, children and adolescents were challenged in their psychosocial adjustment due to the rapid changes in their everyday lives during the COVID-19 pandemic. Children and adolescents were exposed to the socio-political decisions on pandemic containment and had to cope with a high level of stress and uncontrollability. Following the *perseverative cognition hypothesis* (9), the increase in MHC may also be interpreted as a manifestation of worries of children and adolescents during the COVID-19 pandemic.

In terms of resources factors, we found that children and adolescents with more personal resources reported a lower level of MHC. In our study, personal resources include a generally optimistic attitude with a positive outlook toward life and hopeful expectations for the future, self-efficacy with a belief in one’s ability to master challenges, and the perception of sufficient problem-solving skills to deal with difficult situations (46). Given that global crises, such as the COVID-19 pandemic, have a significant impact on societal scenarios and future planning plays a substantial role in adolescence, it is essential to support children and adolescents in shaping their plans and finding a perspective for the future. Therefore, prevention programs with resource-oriented approaches that encompass the living environment of children and young people are necessary. For instance, the Interministerial Working Group “Health Effects of Corona on Children and Adolescents” proposes implementing mental health coaches in schools (64). These coaches would assist children and adolescents dealing with worries and problems, and serve as an accessible first point of contact for further support.

Further, we found that children and adolescents with more familial resources, represented by a positively perceived family climate, reported a lower level of MHC. A positive family climate is characterized by more perceived family interaction and familial support, as well as good family relationships (47). Previous research shows that a parent–child relationship characterized by warmth, closeness, and emotional openness is a relevant protective factor for children and adolescents recovering from stressful life events (65–67). For this reason, it is necessary to implement prevention and intervention programs with system-oriented approaches targeting parents and their children in order to improve family communication and relationships.

Finally, our study demonstrates the importance of social integration of children and adolescents during a global crisis such as the COVID-19 pandemic. Children and adolescents with more perceived social support reported lower levels of MHC. Research on resilience in children and adolescents shows that more perceived social support could reduce the adverse effects of low personal resilience during the COVID-19 pandemic (68). Especially during puberty, children and adolescents begin to orient themselves outside the family and often look to their peers for support (69). Therefore, it is crucial to empower children and adolescents to take on a supportive role and strengthen their peer support. Training programs could be implemented in schools with peer-led interventions (70) or social skill training. Considering that digital platforms are very familiar to children and adolescents, online peer support training could be additionally used to promote children’s and adolescents’ support skills (69).

### 4.3 Strengths and limitations

The strengths of the current study are its longitudinal design and the large population-based sample. Further strengths are the national representativeness of the results and the possibility of monitoring MHC among children and adolescents for more than 3 years. Due to the fact that the study sample is drawn by matching data from the German Microcensus, the results are not generalizable for other countries. It should be noted that a variety of other different health conditions and symptoms, such as fatigue, have been reported in the context of Long/Post-COVID, which are not included in the items of the HBSC SCL. However, using psychometrically sound instruments like the HBSC SCL allows for comparability in research. Lastly, it is important to mention that also other factors might have influenced the frequency of MHC, e.g., other seasonal viral infections and other ongoing socio-political crises.

## 5 Conclusion

Supporting youths in global crises is crucial to protect and maintain their health and well-being. Our study highlights the vulnerability of children and adolescents in a global crisis like the COVID-19 pandemic, identifying key risk factors such as being female, living in single-parent families, or having mentally ill or highly burdened parents. Personal resources, such as an optimistic attitude toward the future and the perception of sufficient problem-solving skills, a good family climate with more perceived familial support and good familial relationships, and social resources with the perception of social support and social inclusion, are significant protective factors. Understanding these risk and protective factors enables the development of targeted prevention and intervention programs. As the prevalence of MHC has increased in recent years, screening for children and adolescents with MHC in pediatric outpatient clinics or other healthcare facilities could help to identify burdened families early in times of global crises.

## Data Availability

The raw data supporting the conclusions of this article will be made available by the authors, without undue reservation.
