# Supplementary material for: Supporting youths in global crises: an analysis of risk and resources factors for multiple health complaints in children and adolescents during the COVID-19 pandemic
Source: Front Public Health. 2025 Feb 13;13:1510355. doi: 10.3389/fpubh.2025.1510355 (PMC11864938; doi:10.3389/fpubh.2025.1510355)
Supplement: Supplementary file 1 [file Supplementary_file_1.docx]

Supplementary Material

# Supplementary Figures

## Supplementary Figure 1


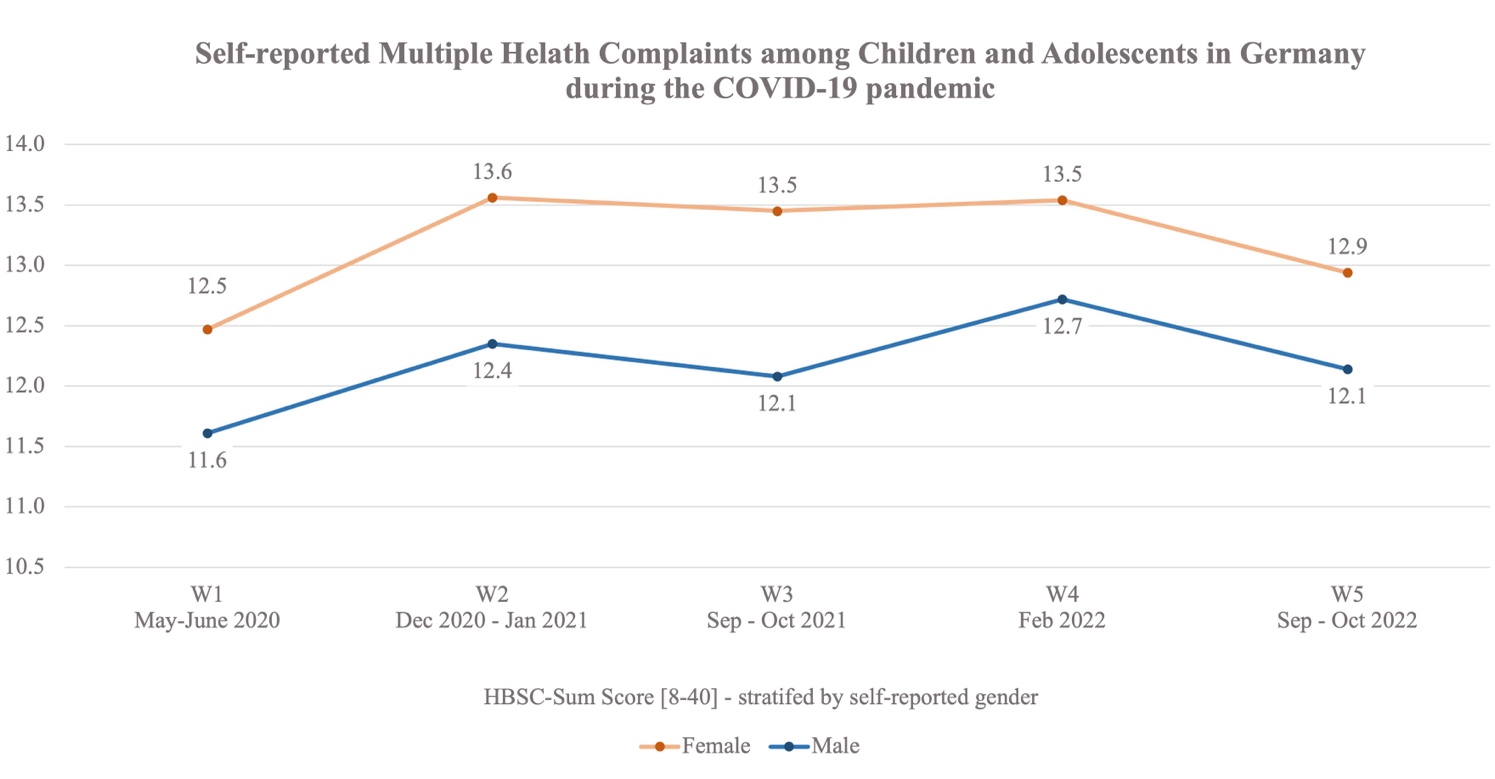


**Supplementary Figure 1:** Self-reported multiple health complaints among children and adolescents in Germany during the COVID-19 pandemic – Sum scores of the HBSC-SCL [8-40] within the five survey waves of the COPSY study, stratified by gender. Higher values indicate more complaints.

## Supplementary Figure 2


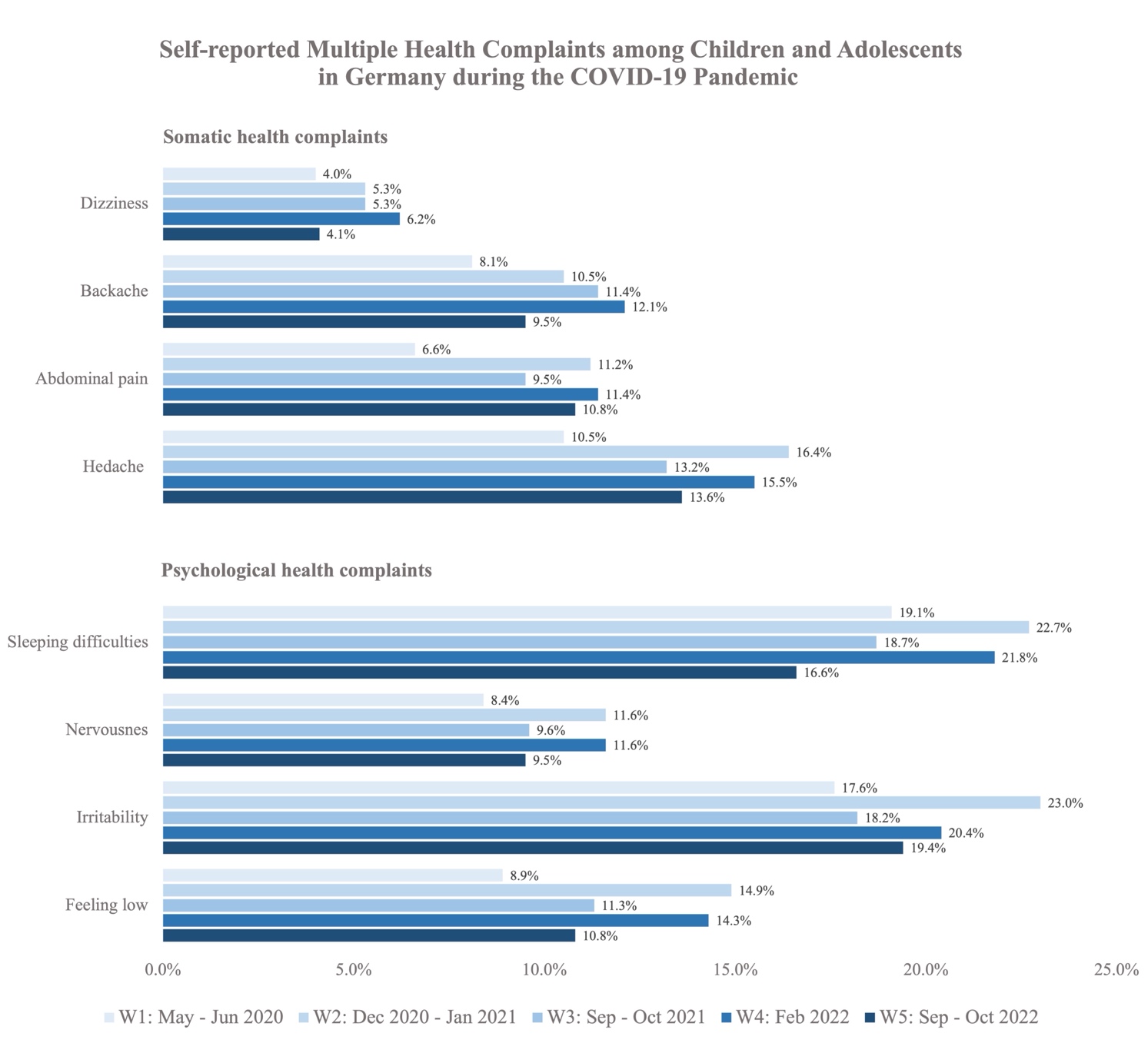


Supplementary Figure 2: Relative frequencies of the eight self-reported health complaints among children and adolescents in Germany within the five COPSY survey waves during the COVID-19 pandemic, divided into somatic and psychological health complaints. The frequencies reflect the percentage of children and adolescents reporting each health complaint at least 3-4 days per week or more often.

## Supplementary Figure 3


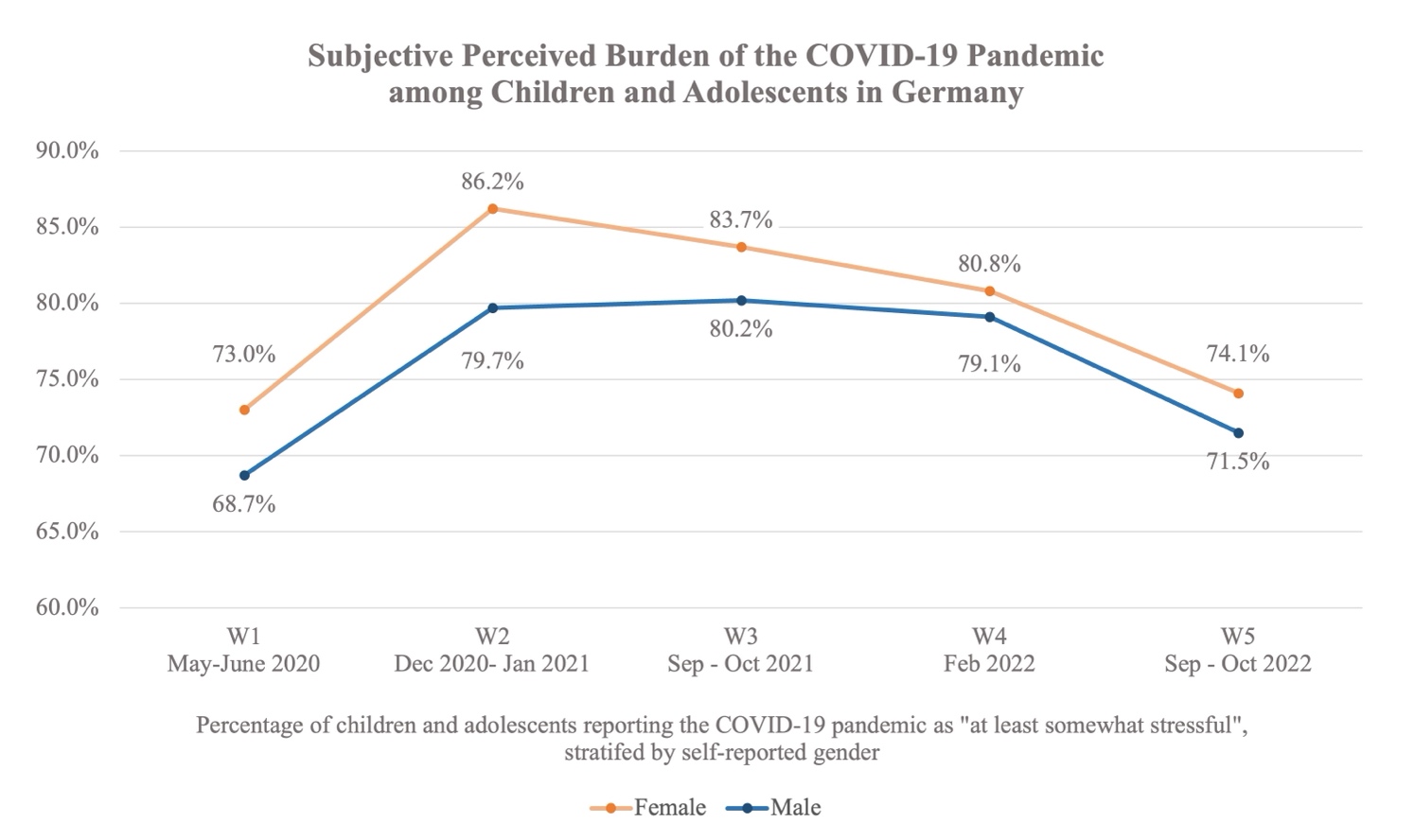


**Supplementary Figure 3:** Subjective perceived burden of the pandemic among children and adolescents during the COVID-19 pandemic in Germany, stratified by self-reported gender. The percentage represents the proportion of children and adolescents who reported the COVID-19 pandemic as "at least somewhat stressful" in each survey wave.

## Supplementary Figure 4


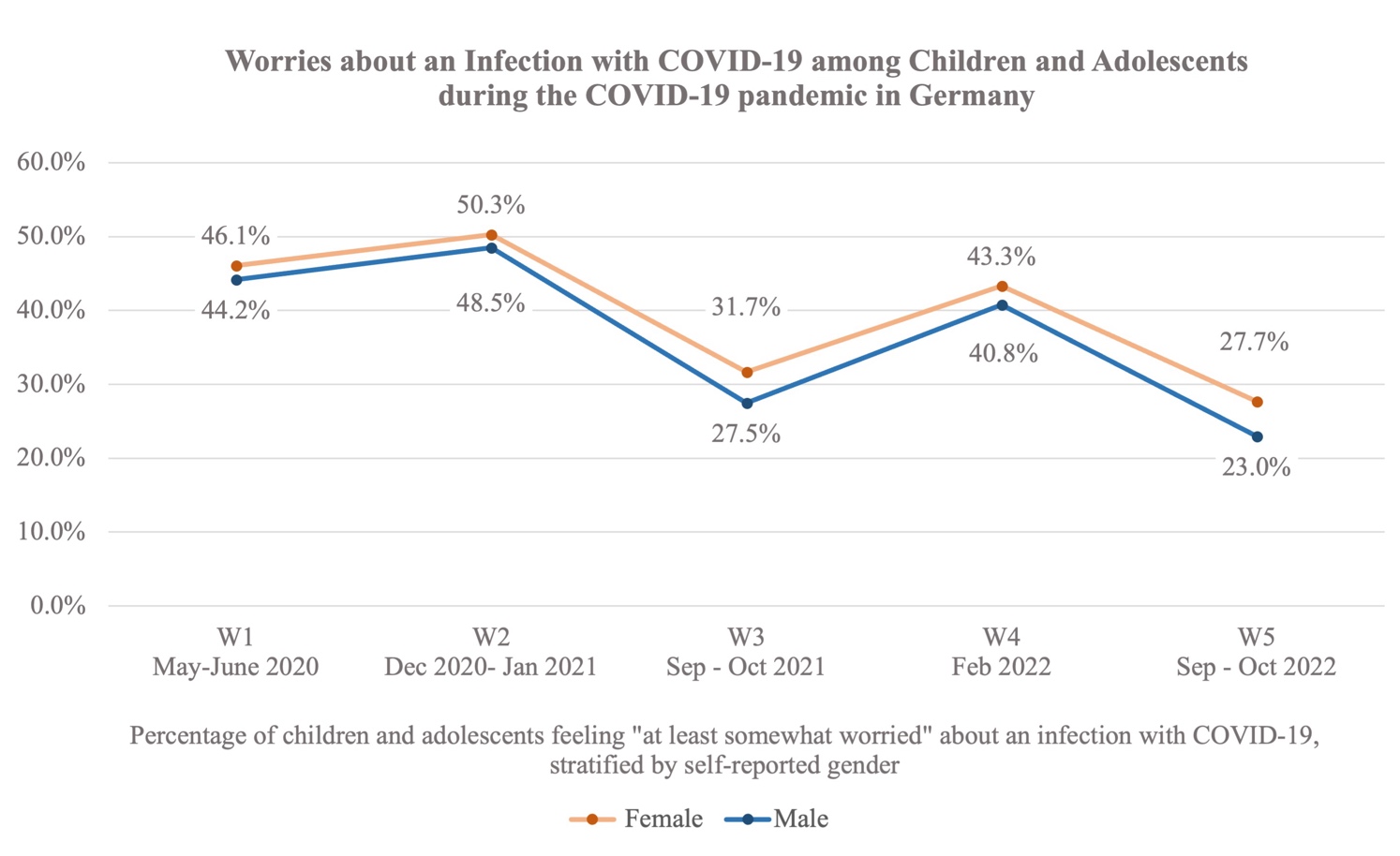


Supplementary Figure 4: Worries about an infection with COVID-19 among children and adolescents during the COVID-19 pandemic in Germany, stratified by self-reported gender. The percentage represents the proportion of children and adolescents who reported being “at least somewhat worried” about an infection at each survey wave.
